# Supplementary material for: Effectiveness and implementation of “Graduation Approach” livelihood programs with displaced populations: A systematic review
Source: medRxiv. 2025 Aug 5:2025.08.01.25332702. Preprint. [Version 1] doi: 10.1101/2025.08.01.25332702 (PMC12340879; doi:10.1101/2025.08.01.25332702)
Supplement: Supplement 1 [file NIHPP2025.08.01.25332702v1-supplement-1.pdf]

Supplemental Table 1. Complete search terms for all reviewed databases

Database(s)	Search String
**EconLit, Academic Search Ultimate, Family & Society Studies Worldwide, and APA PsycINFO**	All searched through EBSCO<br>S1: AB (Refugee* OR migrant* OR "displaced person*" OR "displaced people" OR camp OR camps OR settlement OR shelter OR accommodation OR displacement OR "asylum seeker*") OR SU (refugee OR "refugee camps" OR "human migration" OR "natural disasters" OR "emigrants and immigrants" OR "emigration and immigration") OR TI (Refugee* OR migrant* OR "displaced person*" OR "displaced people" OR camp OR camps OR settlement OR shelter OR accommodation OR displacement OR "asylum seeker*")<br>S2: AB ("ultra poor graduation" OR "upg" OR "targeting the ultra poor" OR "TUP" OR (("graduat*") AND ("poverty" OR "poor") AND ("approach*" OR "program*" OR "model*"))) OR TI ("ultra poor graduation" OR "upg" OR "targeting the ultra poor" OR "TUP" OR (("graduat*") AND ("poverty" OR "poor") AND ("approach*" OR "program*" OR "model*")))<br>S3: S1 AND S2 AND (Published Date: 20020101-20241012)
**PubMed**	#1 (Refugee*[Title/Abstract] OR migrant*[Title/Abstract] OR "displaced person*"[Title/Abstract] OR "displaced people"[Title/Abstract] OR camp[Title/Abstract] OR camps[Title/Abstract] OR settlement[Title/Abstract] OR shelter[Title/Abstract] OR accommodation[Title/Abstract] OR displacement[Title/Abstract] OR "asylum seeker*"[Title/Abstract]) OR (refugees [MeSH Terms] OR "refugee camps" [MeSH Terms] OR "human migration" [MeSH Terms] OR "natural disasters" [MeSH Terms] OR "emigrants and immigrants" [MeSH Terms] OR "emigration and immigration"[MeSH Terms])<br>#2 ((((ultra poor graduation [Title/Abstract]) OR (upg[Title/Abstract])) OR (targeting the ultra poor[Title/Abstract])) OR (TUP[Title/Abstract])) OR ((("graduat*"[Title/Abstract]) AND ("poverty"[Title/Abstract] OR "poor"[Title/Abstract]) AND ("approach*"[Title/Abstract] OR "program*"[Title/Abstract] OR "model*"[Title/Abstract]))))<br>#3 ("2002/01/01"[Date - Publication] : "3000"[Date - Publication])<br>#4 #1 AND #2 AND #3
**Global Health (Ovid)**	#1 (Refugee* or migrant* or "displaced person*" or "displaced people" or camp or camps or settlement or shelter or accommodation or displacement or "asylum seeker*").ab,cw,hw,ot,ti. or (refugee or "refugee camps" or "human migration" or "natural disasters" or "emigrants and immigrants" or "emigration and immigration").sh.<br>#2 ("ultra poor graduation" or "upg" or "targeting the ultra poor" or "TUP" or ("graduat*" and ("poverty" or "poor") and ("approach*" or "program*" or "model*"))).ab,cw,hw,ot,ti.<br>#3 1 AND 2 AND (yr= "2002-Current")
**Web of Science**	#1 TS=(Refugee* or migrant* or "displaced person*" or "displaced people" or camp or camps or settlement or shelter or accommodation or displacement or "asylum seeker*") OR TS=(refugees or "refugee camps" or "human migration" or "natural disasters" or "emigrants and immigrants" or "emigration and immigration")<br>#2 TS=("ultra poor graduation" or "upg" or "targeting the ultra poor" or "TUP" or ("graduat*" and ("poverty" or "poor") and ("approach*" or "program*" or "model*")))<br>#3 1 AND 2 AND (publication date: 2002-01-01 to 2024-10-12)
**Cochrane**	((Refugee* or migrant* or "displaced person*" or "displaced people" or camp or camps or settlement or shelter or accommodation or displacement or "asylum seeker*") OR (refugees or "refugee camps" or "human migration" or "natural disasters" or "emigrants and immigrants" or "emigration and immigration")):ti,ab,kw AND (("ultra poor graduation" or "upg" or "targeting the ultra poor" or "TUP" or ("graduat*" and ("poverty" or "poor") and ("approach*" or "program*" or "model*")))):ti,ab,kw

Supplemental Table 1

Supplemental Table 1. Complete data extraction record

Study ID	Title	Lead author	Year of publication	Name of implemented UPG-type program	Country in which the study was conducted	Start date of implementation	End date of implementation	Comparison group type	Implementation setting	Implementing agency	Notable context features	Intervention details:
Technical and Operational Performance Support (TOPS) Uganda Graduation Randomized Control Trial Associate Award 2022	Endline Report of the Resilience Food Security Activity Graduating to Resilience in Uganda, Cohort 1	Technical and Operational Performance Support (TOPS) Uganda Graduation Randomized Control Trial Associate Award (Antoine Guilhin, Jon Will, Gabriel Dilla, Lucas Kwane, Nathanael Goldberg, and Doug Parkerson)	2022	Graduating to Resilience (Cohort 1)	Uganda	2018 (Cohort 1)	2021 (Cohort 1)	Randomized control group; Other: 2- stage randomization: first by treatment and control village, then by households (allowing "spillover control" and "pure control" groups in treatment and control villages, respectively)	Extremely poor refugee and host households in the Rwamwanja Refugee Settlement and in the surrounding host community of Kamwenge District, which includes the entire Rwamwanja Refugee Settlement as well as four sub-counties and three town councils (TC) contiguous to the settlement for the host community (Nkoma, Bigodi, Bihanga, Bwizi, Nkoma Katalyeba TC, Bigodi TC, and Lyakahungu TC)	AVSI with a consortium including Trickle Up and IMPAQ International	Kamwenge District is in southwestern Uganda and hosts the Rwamwanja Refugee Settlement. This settlement is home to approximately 55,000 refugees, predominantly Congolese, most of whom arrived in or after 2012. The surrounding non-refugee Ugandan population also faces significant development challenges and remains food insecure, up to two-thirds of the population in the Mid-Western sub-region experiences some level of food insecurity.	
IMPEL 2023	Midline Report of the Graduating to Resilience RFSA in Uganda, Cohort 2	Implementation-Led Evaluation & Learning Associate Award (IMPEL)	2023	Graduating to Resilience (Cohort 2)	Uganda	2022 (Cohort 2)	2024 (Cohort 2)	Randomized control group	Refugees and host community in Kamwenge District, Uganda	AVSI with consortium including Trickle Up and American Institutes for Research (AIR)	Study follows up on AVSI Foundation survey findings of high depression prevalence and severity and suicidality in the Palabek refugee settlement in northern Uganda	
Beltramo 2022	Compelling evidence that the graduation approach promotes economic and social integration in displacement settings: the case of Mozambique	Theresa Beltramo & Sandra Sequeira	2022	Maratane graduation program	Mozambique	2019	2021	Other control group	In and around the Maratane Refugee Camp in rural Nampula. The camp is home to about 9,500 refugees and asylum seekers and living around the camp are approximately 15,000 host community individuals who depend on the camp for social services provided by UNHCR and by the government	UNHCR, FAC	Not specified	
Moreno-Sanchez 2021	1. Qualitative evaluation of the Poverty Alleviation Program Produciendo por mi Futuro in Colombia<br>2. Changes in subjective well-being, aspirations and expectations in participants of poverty alleviation programs: A qualitative analysis of Produciendo por mi Futuro in Colombia	1. Rocio Moreno-Sanchez<br>2. Rocio Moreno-Sanchez	1. 2021<br>2. 2021	Produciendo por mi Futuro (PxMF)	Colombia	2015	2017	No comparison (qualitative study)	13 municipalities located in three Colombian departments	Prosperidad Social (PS), Fundacion Capital	Participants were either extremely poor or victims of forced displacement or both these conditions.<br>The setting might suffer from diverse natural disasters.	
UNHCR Costa Rica 2016	Graduation Model Pilot Project 2014-2016. UNHCR Costa Rica	UNHCR Costa Rica	2016	Graduation Model (GM)	Costa Rica	2014	2016	Participant baseline measures		UNHCR, the Association of International Consultants and Advisors (ACAI) (supporting PoC), the House of Rights in Desamparados (HoR) (supporting the women from host community)	Most participants were refugees and asylum seekers, another group of women participants were socially vulnerable and were from Costa Rica (or other countries). The number of nationals vs. vulnerable migrants vs. refugees/asylum seekers is not specified.<br>Most of the population in the project (99%) lived in Costa Rica's metropolitan area.<br>Nationals had a lower level of education than foreign "persons of concern"	

Number of participants	Type of displacement	Intervention inclusion criteria	Intervention exclusion criteria	Household characteristics	How many distinct interventions (different site, components, etc.) were implemented?	Identify INTERVENTION #1:	Identify INTERVENTION #2:	Identify INTERVENTION #3:
11,149 (2,212 in pure control, 2,226 in T1, 2,229 in T2, 2,206 in T3, and 2,202 in spillover control)<br>endline 10,514	Not specified	"Extremely poor refugee and host households" - defined as households that were extremely poor or poor with a woman or youth (between 15 and 35 years old) who are economically active or can be made economically active. (assessed based on a social mapping exercise and household scorecard with wealth ranking)	Not specified	Not specified	3	T1: Standard Graduation	T2: Group Coaching	T3: Empowerment Model
7,000 households (in this review we consider the evaluation "designed specifically around Cohort 2 interventions" (p. 3), not the follow-up survey of Cohort 1 also mentioned in the report).	Not specified	Not specified	Not specified	95% of primary participants are women.	2	"Refined Graduation" Approach including low-cost mental health treatments (group therapy informed by Interpersonal therapy (IPT)); evaluation conducted at MIDLINE, not endline	"Refined Graduation" WITHOUT group therapy; evaluation conducted at MIDLINE, not endline	N/A
150 households, half refugee, half host (approx.), with ~300 controls (not specified how they were selected)	Not specified; refugees from DRC	Not specified	Not specified	Not specified	1	Consumption support, skill training, self- and wage employment support	N/A	N/A
10,000<br>Interviewed: 12 participants from 6 municipalities in 3 departments (1 dropped out before end of surveys); they also interviewed the respective coaches, 5 of these respondents were forcibly displaced	Forced displacement due to the armed conflict	Extreme poor OR victim of forced displacement OR both	Not specified	Among interviewees: 9 in rural area/3 in urban, range of literacy levels and family types and sizes, most own their houses (via inheritance, donations, or purchase), range of land tenure status, most have electricity, variable distance to urban center, sanitation access, and cooking methods	1	Graduation program with two distinct phases	N/A	N/A
Direct: 566 people (including 429 PoC and 139 women from the host community)<br><br>Indirect: 1869 participants (if considering dependents)	General asylum seekers and refugees (not specified); most migrants were Colombian and Salvadoran, with some Nicaraguan, Honduran, and Venezuelan, and a few from other countries in Latin America and Africa	refugee or asylum seeker, Costa Rican socially vulnerable women, other socially vulnerable women migrants.	Not specified	Demographics are described but other target characteristics are not given. Only a couple of adolescents were included due to exceptional circumstances (not targeted to youth).	1	The methodology applied in Costa Rica has been a combination of different interventions related to social assistance, livelihoods support and microfinance. The project provided personalized coaching, cash transfers to enable them to meet basic needs, training, access to health services, and access to valid documentation, among others. Based on the principal of promoting self-reliance, the following interventions were implemented: home visits/social and livelihood assessments, socio-economic assessments, psycho-social support, assistance with an action plan for local integration in the country and comprehensive casework. The counsellors involved are Social Work and Psychology professionals.<br>The Model seems to have two tracks: self-employment / wage-employment<br><br>Note that the results reported derive ONLY from the 116 "graduated" cases with complete file information.<br>The baseline results also represent this complete case sample.<br>The CONTROL group documents the BASELINE data of the ENTIRE PARTICIPANT POPULATION	N/A	N/A

Program components (INTERVENTION #1)	Duration and sequencing of components (INTERVENTION #1)	Adaptations (INTERVENTION #1)	Program components (INTERVENTION #2)	Duration and sequencing of components (INTERVENTION #2)	Adaptations (INTERVENTION #2)	Program components (INTERVENTION #3)	Duration and sequencing of components (INTERVENTION #3)	Adaptations (INTERVENTION #3)	Study design
Social protection (consumption support), Livelihood promotion (asset transfer), Financial inclusion (savings), Social empowerment (coaching), Other: Linkages and referrals	-Consumption support: $15/refugee household or $6/host household per month x 12 months<br>-Coaching: 1-hour individual coaching sessions (weekly at first, reduced to biweekly) throughout the duration of the program (Feb 2019-Jan 2021), according to structured curriculum, individual coaches managed 25 households on average<br>-Nutrition screening and referrals<br>-Core and technical skills training: aimed to help select a livelihood activity, and then plan and manage it effectively to develop business skills (weekly, seasonal Farmer Field/Business School meetings, Business Coaching delivered quarterly to participants at their business site, and weekly Financial Literacy Training conducted during VSLA meetings)<br>-Village Savings and Loan Association (VSLA) activities: weekly [illegible] activities to save money, take loans, and contribute to self-insurance/welfare fund<br>-1-time cash transfer of $300, 7 months after start of project<br>-Linkages and referrals, including market event, access to critical services tackling issues such as nutrition, gender-based violence, health and WASH	-Weekly coaching meetings were reduced to biweekly during implementation after realizing the participants could not keep up with such frequent meetings.<br>-Individual coaching was conducted by phone call during COVID	Social protection (consumption support), Livelihood promotion (asset transfer), Financial inclusion (savings), Social empowerment (coaching), Other: Linkages and referrals	Same as above, except coaching was conducted weekly in 2-hour sessions with an average of 25 participants per group	Due to COVID, the sessions were carried out remotely via conference calls of 5 people in the later stages.	Social protection (consumption support), Financial inclusion (savings), Social empowerment (coaching), Other: Linkages and referrals	Same as T1, except T3 did NOT receive the asset transfer	Same as T1	Randomized controlled trial, Economic evaluation
Social protection (consumption support), Livelihood promotion (asset transfer), Financial inclusion (savings), Social empowerment (coaching), Other: Group therapy for women participants to share challenges, discuss problem solving, and offer mutual support	-A small cash transfer of $4-5 per household member per month, x 12 months<br>-A lump sum cash transfer of around $300 6 months after the start of the program<br>-Technical trainings in financial literacy, selecting an enterprise, planning, management, agricultural skills, and bank linkages during the first 6 months of the program (before the asset transfer)<br>-Coaching on health, nutrition, gender, life skills, and sanitation (48 sessions over 24 months + 8 quarterly one-on-one "touchpoints"). Coaches also provide referrals and linkages to public and private sector services as needed.<br>-Savings and loan associations (duration and timing not specified)<br>- Group therapy: 12 sessions across 3 months. Each quarter, participants were intended to have 6 group coaching sessions and at least 1 individual touch point.	The intensity of the coaching component was adapted compared to cohort 1, they adopted a "hybrid" coaching approach relying mostly on group coaching sessions every 2 weeks, with "quarterly individual touchpoints" for one-on-one participant followup with coaches.	Social protection (consumption support), Livelihood promotion (asset transfer), Financial inclusion (savings), Social empowerment (coaching)	Same as above, but without the group therapy component	Same as above, but without the group therapy component	N/A	N/A	N/A	Randomized controlled trial
Social protection (consumption support), Livelihood promotion (asset transfer), Social empowerment (coaching)	Consumption support to provide a basic standard of living as well as cash transfers for transport and investment in productive assets related to the employment track or sector selected by participants.<br>Skills training including those related to resume writing, development of soft skills, language and financial literacy, market-oriented skills and vocational training, as well as coaching services to provide encouragement, build self-esteem and to personalize interventions to individual needs.<br>Facilitation with self and wage employment, including paid apprenticeships to improve linkages to jobs, which helps improve the employability of participants with limited experience. All those benefitting from self-employment support also receive personal coaching on the design of a business plan.	Paid apprenticeships thought to improve job linkages and prospects for people with low experience	N/A	N/A	N/A	N/A	N/A	N/A	Non-randomized experimental study
Livelihood promotion (asset transfer), Financial inclusion (savings), Social empowerment (coaching)	Phase 1 (8 months): Home visits - 7 personalized visits by coach for training activities with tablet app modules, 3 group workshops, presentation of business plan to project and government officials<br>5-month transition time: program structural changes, no intervention<br>Phase 2 (10 months):<br>"Pre capitalization:" 5 workshops (including savings group training) and 2 household visits, development of business plan, present project for approval or fund disbursement<br>Disbursement of startup capital<br>"Monetary incentive": 500 US dollars to invest in the establishment or improvement of a productive entrepreneurship<br>"Post-capitalization:" 5 home visits supported app-based financial education called USTA and 4 additional workshops, suppliers' fair for market integration (graduation ceremony)	Study 1:<br>Potential adaptation for participants' education level. It is worth highlighting that elements such as the coach and the tablet and its modules do not require minimum levels of education, as happens with the work logbook, profile design, and the guides, whose development require at least basic reading and writing skills.<br>Study 2:<br>Among other factors of personal development mentioned by the participants, we highlight their feelings of tranquility and happiness	N/A	N/A	N/A	N/A	N/A	N/A	Qualitative research
Social protection (consumption support), Livelihood promotion (asset transfer), Financial inclusion (savings), Social empowerment (coaching)	Personalized coaching<br>Cash transfers to meet basic needs<br>Training<br>Access to health services<br>Access to valid documentation<br>Home visits/social and livelihood assessments<br>Psychosocial support<br>Action plan for local integration in the country<br>Comprehensive casework<br>Economic assistance: seed capital for self-employment) (average value 257,226 CRC), housing, food, both housing and food, education, technical training and materials, private sector health services, transportation subsidy, cash assistance to renew ID<br><br>The above are listed but not further specified in terms of details or timing.<br><br>Each person has different duration time until graduation. 4.0% 0-6 months, 16.0% 7-12 months, 41.2% 13-18 months, 31.4% 19-24 months, 5.7% 25-30 months, and 1% 31-36 months.	A total of 42 cases were included in the project already complying with the income graduation criteria (294,000), because at the beginning of the project UNHCR included some "fast track" cases who only needed a punctual support to achieve a stable livelihood	N/A	N/A	N/A	N/A	N/A	N/A	Other: Pre-post design for each cohort

Intervention and Comparisons:	UPG intervention #1 Food security	UPG intervention #1 Employment	UPG intervention #1 Living conditions/quality of life	UPG intervention #1 Social wellbeing	UPG intervention #1 Poverty	UPG intervention #1 Dependency on humanitarian aid
	Food security index Z-score: 0.63*** (0.04)<br><br>(Food security index is a z-score index of three components (Food Consumption Score (FCS), Negative Household Food Insecurity Access Scale (HFIAS), and Length/height-for-age (HAZ) for children under 5), standardized to a mean of 0 and SD of 1 in the Pure Control.)	Total time spent working: 3.07*** (1.14)	Basic drinking water: 0.05** (0.02)<br><br>Handwashing with soap on premises: 0.13*** (0.02)<br><br>Access to basic sanitation service: 0.03 (0.02)	Subjective well-being index Z-score: 0.62*** (0.05)<br><br>Subjective well-being index is a z-score index of two components (Negative Kessler 6 score and Life satisfaction from 1 to 10)	Value of productive assets: 520.54*** (77.67) PPP $ greater than pure control group<br><br>Monthly income: 43.30*** (7.34) PPP $ greater than pure control group<br><br>Monthly consumption: 24.53*** (3.12) PPP $ greater than pure control group	N/A
	N/A	N/A	N/A	Group therapy ("In host community") scored 0.22 SDs lower on optimism and 0.14 SDs lower on social wellbeing indices, relative to the regular intervention group.<br><br>(Note: "The optimism index aggregates information about personal aspirations and aspirations for the participant's children. The social wellbeing index aggregates information about subjective social status and subjective social worth.")	The therapy group ("in host community") scored 0.12 SDs lower than the regular intervention group on "economic activity index" of information about participant labor supply, investment, and intention to expand economic activity; group differences driven by difference in investment in income-generating activities	
	By the end of the program, 40 percent reduction in the probability of skipping meals compared to control	N/A	Resulting from cash transfers equivalent to 24-40 months of household income, more than 10% of participants have invested in buying or constructing a new house, and more than 10% have invested in electrical grid connections.	Self reported significant increases in levels of trust in the other groups compared to non-participants at the end of the programme.	Graduation participants have 1.3 times more household income compared to those in the control group at the end of the programme<br>At the end of the programme, graduation participants report savings on average of $14/month, versus control participants who reported no savings.	N/A
	8 out of 11participants reported positive changes in their households' food security during the program (quantity consumed, frequency of consumption, diversified foods), due to consumption of food produced or sold, or to factors external to the program. 2 participants reporting no changes were not in a situation of scarcity before the intervention.<br>Coaches could not assess participant changes.	All participants acquired productive assets (some received from other development programs or used their own resources)<br>7 participants already reinvested profits from productive activity; coaches confirm increase in productive assets beyond those distributed by program due to business training, savings and investment training, coaching, and expectations linked to financial support<br>4 of the "dependent activity households" increased independent activity participation<br>7 participants diversified their productive activities during program implementation and 3 reported no change	6 out of 11 participants made refurbishments, extensions or renovations to their houses.<br>Findings were confirmed by coaches.	Study 1:<br>Access to formal basic health services has improved or remained stable for most participants (all part of subsidized health regime)<br>All reported improved self-esteem, self-value, self-confidence, some changed in aspirations and expectations; those with worse socioeconomic conditions improved more in subjective well-being<br>Coaches confirm personal development changes, attribute to workshops and home visits, particularly role of tutor<br>Participants with severe socioeconomic limitation and low education, as well as women who survived mistreatment or violence, had high achievement in "personal development goal"<br>Study 2:<br>Coaches specify global improvements in: self-confidence, no longer feeling shy, improved self-esteem, self-value, respect for themselves and their families, improved public speaking and articulation of ideas, improved personal care and presentation, improved family life, and concrete goals and purposes.<br>Changes in agency and locus of control: 10 participants showed increases in agency; 2 had internal locus of control, 2 external, 3 tend towards internal, and 3 tend towards external.	1. 6/11 participants increased spending on food<br>3/11 increased relative spending on clothing, shoes<br>2/11 increased spending on recreation (1 reduced)<br>1 increased spending on education due to children's needs<br>2 reported increases in spending on cleaning products<br>3 spent more on health because of shocks<br>7 acquired cell phones<br>6 increased household appliances<br>All participants saved and reinvested earnings in productive enterprise. 8 had positive change in savings, 3 had no change in saving habits. 3 continue saving in groups, 2 in money boxes, 2 by lending, and 5 save to reinvest; coaches attribute increased saving to financial education, app use, and tutor support<br>9 participants had improved debt management and 3 attributed this to the cash incentive. 2 had negative changes, 1 had no change; coaches unclear on debt management improvements but highlight the house visits and app as a first step; participants with fewer limitations and higher education had better debt management achievements.<br>Coaches: spending fundamentally increased for clothing, shoes, cleaning, beauty products due to personal development and increased income, some noted no changes in consumption<br>Coaches generally confirmed an increase in domestic assets due to trainings on saving/debt, enterprise earnings, and personal development training	N/A
	N/A	2014- 4.0% unemployed<br>2015- 4.3% unemployed<br>2016 cohort- complete data not available	91.2% finished project holding valid refugee documentation (44.1% received economic assistance from UNHCR or partner to do so)	57.0% reported emotional wellbeing had improved<br>25.4% reported no change<br>13.3% reported worse emotional wellbeing<br>4.4% no answer<br><br>82% do not participate in organized groups.	78.9% of households reached monthly income >= national minimum wage by the end of the project<br>16.7% did not reach threshold<br>4.4% no answer<br><br>3.5%- no reported income<br>13.2%- 1000-100,000<br>39.0%- 100,001-250,000<br>39.3%- 250,001-500,000<br>4.4%- >500,000<br>4.4%- no answer<br><br>67.5% income increased<br>18.4% income decreased<br>9.8% income maintained<br>4.4% no answer<br><br>32.4% do not save money<br>21.2% save 1000-20,000 CRC monthly<br>14.2% save > 20,000<br><br>Reported increase in access to bank accounts and financial services.	N/A

UPG intervention R1 Mental health	UPG intervention R2 Food security	UPG intervention R2 Employment	UPG intervention R2 Living conditions/quality of life	UPG intervention R2 Social wellbeing	UPG intervention R2 Poverty	UPG intervention R2 Dependency on humanitarian aid	UPG intervention R2 Mental health	UPG intervention R3 Food security	UPG intervention R3 Employment	UPG intervention R3 Living conditions/quality of life	UPG intervention R3 Social wellbeing	UPG intervention R3 Poverty	UPG intervention R3 Dependency on humanitarian aid	UPG intervention R3 Mental health	Participant baseline (if applicable) Food security	Participant baseline (if applicable) Employment
N/A	Food security index Z-score: 0.63*** (0.04)	Total time spent working: 2.07 (1.44)	Basic drinking water: 0.03 (0.02)<br><br>Handwashing with soap on premises: 0.14*** (0.02)<br><br>Access to basic sanitation service: 0.02 (0.02)	Subjective well-being index Z-score: 0.55*** (0.06)	Value of productive assets: 424.53*** (69.28) PPP $ greater than pure control group<br><br>Monthly income: 44.57*** (7.02) PPP $ greater than pure control group<br><br>Monthly consumption: 25.77*** (3.24) PPP $ greater than pure control group	N/A	N/A	Food security index Z-score: 0.51*** (0.04)	Total time spent working: 2.52* (1.35)	Basic drinking water: 0.03 (0.02)<br><br>Handwashing with soap on premises: 0.11*** (0.01)<br><br>Access to basic sanitation service: 0.01 (0.02)	Subjective well-being index Z-score: 0.44*** (0.06)	Value of productive assets: 233.50*** (73.00) PPP $ greater than pure control group<br><br>Monthly income: 30.39*** (6.71) PPP $ greater than pure control group<br><br>Monthly consumption: 17.11*** (3.12) PPP $ greater than pure control group	N/A	N/A	HDDS score at baseline for treatment villages = 3.06 (SD=1.29), for control villages = 3.06 (SD=1.39)	Mean value for employment in the past 12 months at baseline in treatment villages = 0.57 (SD=0.47), in control villages = 0.57 (SD=0.47)<br><br>Mean value for having a business activity in the past 12 months at baseline in treatment villages = 0.25 (SD=0.44), in control villages = 0.25 (SD=0.43)
The therapy group ("in host community") scored 0.11 SD higher on "aggregate measure of psychological distress" (a combined, normalized measure incorporating Kessler-10, PHQ-9, and GAD-7) than the regular intervention group. No significant effect was found in the refugee community.<br>The effects are concentrated among participants with high rates of baseline distress.	N/A	N/A	N/A	N/A	N/A	N/A	N/A	N/A	N/A	N/A	N/A	N/A	N/A	N/A	N/A	N/A
N/A	N/A	N/A	N/A	N/A	N/A	N/A	N/A	N/A	N/A	N/A	N/A	N/A	N/A	N/A	N/A	N/A
N/A	N/A	N/A	N/A	N/A	N/A	N/A	N/A	N/A	N/A	N/A	N/A	N/A	N/A	N/A	Some participants had no food security limitations, others had moderate limitations in the past or at the time of intervention, and others had severe limitations.	High heterogeneity reported in terms of productive activities. 5 households had "dependent productive activity" before the intervention
26.5% of participants (or household member) received counselling	N/A	N/A	N/A	N/A	N/A	N/A	N/A	N/A	N/A	N/A	N/A	N/A	N/A	N/A	N/A	2014- 37.9% unemployed<br>2015- 33.4% unemployed<br>2016- 29% unemployed

Participant baseline (if applicable) Living conditions/quality of life	Participant baseline (if applicable) Social wellbeing	Participant baseline (if applicable) Poverty	Participant baseline (if applicable) Dependency on humanitarian aid	Participant baseline (if applicable) Mental health	Control group (if applicable) Food security	Control group (if applicable) Employment	Control group (if applicable) Living conditions/quality of life	Control group (if applicable) Social wellbeing	Control group (if applicable) Poverty	Control group (if applicable) Dependency on humanitarian aid	Control group (if applicable) Mental health	Implementation factors:
N/A	Subjective well-being index at baseline for treatment villages = -0.01 (SD=1.01), for control villages = 0.01 (SD=1.00)		N/A	N/A	Spillover control, food security index Z-score: 0.08* (0.05)<br>----<br><br>Comparison/reference value of pure control, food security index Z-score: 0 (1)	Spillover control, total time spent working: 0.13 (1.32)<br><br>----<br><br>Comparison/reference value of pure control, total time spent working: 33.87 (SD= 34.45)	Spillover control, basic drinking water: 0.01 (0.02)<br><br>Spillover control, handwashing with soap on premises: 0.05** (0.01)<br><br>Spillover control, access to basic sanitation service: -0.01 (0.02)<br><br>This table (p. 73) is missing the "mean of pure control" row for reference, but it is available for the disaggregated refugee/host figures on pp. 74-75	Spillover control, subjective well-being index Z-score: 0.07 (0.05)<br><br>----<br><br>Comparison/reference value of pure control, subjective well-being index Z-score: 0 (1)	Spillover control, value of productive assets: 29.23 (72.13) PPP$ greater than pure control group<br><br>Spillover control, monthly income: 6.17 (6.29) PPP $ greater than pure control group<br><br>Spillover control, monthly consumption: 6.14* (3.70) PPP $ greater than pure control group<br><br>----<br><br>Comparison/reference value of pure control, value of productive assets: 556.50 PPP$ (SD= 1,532.00)<br><br>Comparison/reference value of pure control,	N/A	N/A	
N/A	N/A	N/A	N/A	N/A	N/A	N/A	N/A	N/A	N/A	N/A	N/A	
comparator	N/A	N/A	N/A	N/A	comparator	N/A	N/A	comparator	Zero savings each month at the end of the programme.	N/A	N/A	
Some had no limitations in housing conditions, others had current and past limitations, others lived in moderate housing conditions with some improvements since childhood, and others had past limitations with more recently improved conditions (current moderate limitations or without present limitations)	Some participants had no/minor limitations in access to health services currently or in childhood/adolescence, some had moderate access and others moderate limitations, others had severe limitations.	Some participants had no limitations in terms of asset holding, others severe past limitations, others severe permanent limitations, and others moderate limitations.	N/A	N/A	N/A	N/A	N/A	N/A	N/A	N/A	N/A	
N/A	77% do not participate in organized groups.	At intake (average across cohorts):<br>15.8%- no reported income<br>29.1%- 1000-100,000<br>41.2%- 100,001-294,000<br>11.4%- 294,001-500,000<br>3.5%- >500,000<br>(benchmark = 294,000)<br><br>58.% do not save money	N/A	N/A	N/A	54.1% had paid job -- 16.1% self employed, 17.2% occasionally employed, 13.4% permanently employed, 5.4% temporarily employed<br><br>43.2% unemployed	N/A	N/A	30.5% no income<br>32.7% earn ~$150<br>29% earn ~$510<br>42 cases already had "graduation level" income (294,000 local currency) so "fast track" cases needing only minor supports.	N/A	N/A	

UPG intervention Barriers	UPG intervention Facilitators	UPG intervention Feasibility indicators	UPG intervention Sustainability indicators	Overall notes and important considerations
Infrastructural challenges (poor roads, heavy rains, large distances between households, COVID-19 restrictions such as evening curfew)	T3 is $[illegible] cheaper than T1 due to the absence of cash asset transfer. T2 is $[illegible] cheaper than T1 due to group coaching sessions.<br>T2 is 13% cheaper than T1.<br><br>Since the costs for group coaching are lower than those of individual coaching and because the point estimate for T2 (group coaching) is larger than for T1 (individual coaching), T2 has higher estimated cost-effectiveness. Considering the cost-effectiveness of the asset, the treatment groups with the cash asset transfer (T1 and T2) have higher estimated cost-effectiveness than T3 because the benefits advantage of the with-asset groups is proportionally larger than the cost advantage of T3.	All groups: most important reason for participant dropout was households leaving their villages (more so among refugee community, as many returned to DRC); second-most important reason was households losing interest in activity<br><br>Dropout rates were highest in T3 (no asset group) and lowest for T2 (asset transfer with group coaching)	N/A	Results described are *statistically significant at 10% level, **at 5%, ***at 1%. Standard errors given in parentheses. Quantities reported in intervention groups are point estimates of the DIFFERENCES between the groups and 'pure control.'<br><br>Authors note that "treatment effect estimates are larger for T1 and T2 relative to T3, and effects are larger in the host sample than in the refugee sample" (p. 31)<br><br>More detail is available regarding differences in specific kinds of productive assets between groups (Table 12, p. 32), in types of income (Table 13, pp. 32-33), type of monthly consumption (Table 14, pp. 33-34), food security index components (Table 15, pp. 34-35), and subjective well-being index components (Table 16, pp. 35-36). Multiple secondary outcomes are also assessed.
N/A	N/A	N/A	N/A	The authors interpret these findings to mean that "economic activity is significantly associated with psychological distress" and that the group therapy or its implementation was not effective in this context. They don't speculate why this is so, nor do they describe the therapy intervention or its implementation in much detail.<br><br>Quantified findings are reported for the host community only, the brief mentions null findings for the refugee community but does not quantify these.
N/A	N/A	N/A	N/A	In general, a lot of the "study design" details and the implementation details are missing. Even the intervention for the control condition is missing. This makes it difficult for us to compare their effectiveness.
Study 1:<br>Shocks throughout life and during intervention were reported: during the intervention, these included illness, child school dropout, police involvement with family, debt, climate shock (drought), domestic violence, job loss, death in family, extreme rainfall ("external or uncontrollable factors" (p. 112)<br>Household size may reduce magnitude of beneficial effect<br>Men tended to experience lower results<br>Participant physical isolation from urban centers limited market integration<br>Inconsistency of design and approach: "the rules of the game must not change during program execution" (p. 120)<br><br>Study 2:<br>"Adjustment phase" without program activities or communication led to "sense of abandonment and mistrust" (p. 37)<br>Factors presumed to affect educational and work aspirations: household economic instability, lack of food, teenage pregnancy<br>Obstacles mentioned by participants that impede them from fulfilling their aspirations: a lack of work, health problems, death of family members, failure of productive enterprise, not being able to expand the productive activity, a lack of specialized business management training, competition, damage of machinery, not having favorable markets for farming activities, flooding due to extreme rainfall, depression or sadness, corruption in the government programs or the non-reception of reparation for their conditions as victims, conflicts inside the household or with the partner, a lack of land, a lack of safety in the areas in which they live (the presence of criminal groups or other armed groups), migration of people from Venezuela, and the impossibility to have children.	Study 1:<br>Additional pig-rearing cycle sponsored by program would allow improved household nutrition<br>Coaches: WFA food security program implemented simultaneously to PVMP improved food frequency and diversity<br>In households where both members of a couple were formal project participants, productive assets were higher<br>Coaches: productive activities must generate sufficient income to facilitate improved debt management<br>Personal development improvements would be greater if team had sufficient time to not rush the last phase of activities<br>Better results among participants with less extreme poverty and "who in complete, young, and small households which very probably enjoyed double intervention support" (p. 112)<br>Strong "family-based social capital is fundamental when it comes to dealing with shocks" (p. 115)<br>Visits and workshops understood to address different kinds of support and should not be combined to save money<br><br>Study 2:<br>Factors presumed to affect educational and work aspirations: road quality, distance from educational centers, municipality education quality	Study 1:<br>Climate shocks especially detrimental to food security (directly reduces food/leads to loss of livestock, or reduces income for business supply purchases)<br>Forcibly displaced participants had lower changes in result variables, but had better socioeconomic conditions than other participants who were "targeted for poverty" (p. 112) yet "take better advantage of the program" (p. 113)<br>Suggest shifting targeting criteria or program elements to suit needs of heterogeneous participants<br>The coach is central to most program goals, need to manage caseload, reduce overburden, and manage task allocation to enable them to use time efficiently<br>App was viewed favorably and preferable to paper-based materials in a population of low literacy<br><br>Study 2:<br>Some domains of family wellbeing (e.g., children's higher education) require external government support to facilitate; participants identify this as essential	Households moving to capital cities may have had higher specialized health care costs that impeded access<br>Land tenure important for sustainability of agricultural activities<br>Important to assess context-specific risks and shocks and account for these in program design in order to maintain benefits in long term<br>Program must incorporate environmental shock preparedness and adaptation<br>Productive activities could be promoted which emphasize participant interreliance to minimize redundancy and competition in restricted markets<br>Graduation programs should be managed by governments as poverty reduction schemes to ensure continuity and consistency of implementation	Ultimately, participants had heterogeneous profiles in terms of key outcomes at the end of the study and in terms of trajectories--but these weren't discernibly related -> this can relate to the equity issue in terms of program effectiveness<br>Changes in variables are assessed "over time", that is, between childhood/adolescence, an intermediate period, and the beginning of implementation<br>They appear to have done something like a qualitative version of a latent class analysis--lots of "quantizing" qualitative data<br>Highlight the importance of element complementarity to achieve effects<br>Clearer definitions and measurements about outcomes compared to other studies, supported by study 2.
Shift in Salvadoran vs. Colombian population required adjusting operations, as activities had been targeted to a majority Colombian population identified at first	N/A	250 (27.5%) were "excluded from the initiative" (p. 9) due to non-commitment, leaving the country without notice, or losing contact with organization<br><br>A higher percentage of refugee participants (35.5%) than local community participants (22.7%) graduated from the program--attribute to differences in vulnerability among local women and some differences in the intervention	Time scale for implementation was primarily 13-24 months (72.8% of total graduated participants)	It sounds like they adopted a cohort model with perhaps a 1-year implementation cycle, but this isn't stated outright<br>"Endline" by year/time of graduation for each presumed cohort group<br><br>I am disregarding the host community data as these are reported separately and are not as relevant to the review. (I agree though I did extract them!)<br><br>194 participants "graduated" from project as of January 2017<br>199 participants still active<br>19 (3.3%) dropped out<br>250 (27.5%) were "excluded from the initiative" (p. 9) due to non-commitment, leaving the country without notice, or losing contact with organization<br><br>Graduation = 3/4 criteria (#1 compulsory):<br>1. Economic self-sufficiency (can cover basic needs through income generating activity, gross minimum income ~$520 USD per household monthly, saving at least 2-5% of added monthly income<br>2. Increased self-confidence<br>3. Enabled to build support networks in community and social environment<br>4. Carries valid documentation
